# Description of three new species of *Benedictus* (Coleoptera, Chrysomelidae, Galerucinae, Alticini) from China, with comments on their biology and modified ethanol traps for collecting flea beetles

**DOI:** 10.3897/zookeys.1177.102811

**Published:** 2023-08-30

**Authors:** Yongying Ruan, Alexander S. Konstantinov, Albert F. Damaška, Lihao Zheng, Jun Chen, Ziye Meng

**Affiliations:** 1 Plant Protection Research Center, Shenzhen Polytechnic, Shenzhen 518055, China Plant Protection Research Center, Shenzhen Polytechnic Shenzhen China; 2 Systematic Entomology Laboratory, USDA, Smithsonian Institution, P.O. Box 37012, National Museum of Natural History, Washington, DC 20013-7012, USA Systematic Entomology Laboratory, USDA, Smithsonian Institution Washington United States of America; 3 Department of Zoology, Faculty of Science, Charles University, Viničná 7, 128 00 Prague, Czech Republic Charles University Prague Czech Republic; 4 Guang’an Vocational and Technical College, Guang’an 638000, China Guang’an Vocational and Technical College Guang’an China; 5 Key Laboratory of Zoological Systematics and Evolution, Institute of Zoology, Chinese Academy of Sciences, Beijing 100101, China Institute of Zoology, Chinese Academy of Sciences Beijing China; 6 Research Center of Buckwheat Industry Technology, Guizhou Normal University, Guiyang, Guizhou, 550025, China Guizhou Normal University Guizhou China

**Keywords:** Diversity, flea beetles, leaf litter, pan trap, pitfall trap, taxonomy

## Abstract

The diversity and biology of the moss and leaf litter-inhabiting flea beetles are still poorly known. In this study, three new species of *Benedictus* are described from China: *Benedictusfuanensis* Ruan & Konstantinov, **sp. nov.**, *Benedictusquadrimaculatus* Ruan & Konstantinov, **sp. nov.**, and *Benedictuswangi* Ruan & Konstantinov, **sp. nov.** Comments on their biology are given. *Benedictusquadrimaculatus* has a highly unusual morphological feature not reported before in flea beetles: black spots on the abdominal tergites that are visible through the elytra. Traditional and modified ethanol traps were tested and proven useful for collecting leaf litter- and moss-inhabiting flea beetles. Based on our tests, eight traps could collect one specimen each day in the testing sites in Fujian Province; three traps could collect one specimen each day in the testing sites in Guangdong Province.

## Introduction

*Benedictus* Scherer, 1969 consists of 26 species prior to this study, of which eight species are known from China. *Benedictus* species occur in Oriental Region and Papua New Guinea, and the adults are usually wingless and inhabit moss cushions and leaf litter (Sprecher-Uebersax et al. 2009). The most recent studies on *Benedictus* include the taxonomic revisional work by Sprecher-Uebersax et al. (2009) which reported 25 species of the genus, and the description of a new species by Damaška and Aston (2019). *Benedictus* is morphologically allied to *Microcrepis* Chen and *Loeblaltica* Scherer (Sprecher-Uebersax et al. 2009). Recent molecular phylogenetic studies (Damaška et al. 2022; Douglas et al. 2023) revealed that flea beetles from multiple and often distant lineages adapted to moss and leaf litter habitats. Damaška et al. (2022) revealed that *Benedictus* belongs to the *Manobia* generic group, which contains the moss-inhabiting genus *Benedictoides* and leaf surface-living genera *Aphthonoides* Jacoby, *Manobia* Jacoby, and *Phyllotreta* Chevrolat. Despite the studies mentioned above, the true diversity and the biology of the moss-inhabiting species of *Benedictus* are still poorly known. In this work, we describe three new species from China and provide insights into their biology. We also provide a key to the 11 species occurring in China. The feeding habit and living environment of *Benedictusfuanensis* sp. nov. are found to be very similar to that of *Cangshanalticafuanensis*Ruan et al. (2022). In Fujian province, they were found in the same location and share the same host plant *Hypnumplumaeforme* Wilson (Hypnaceae). When reared in the laboratory, they both feed on the distal ends of moss branches of the host plant.

Moss and leaf litter-inhabiting flea beetles are usually collected by the traditional Berlese funnel (e.g., Konstantinov et al. 2013; Linzmeier and Konstantinov 2020; Konstantinov and Linzmeier 2020) and the fan-driven Berlese funnel (Ruan et al. 2020). In Damaška and Konstantinov (2016), specimens were collected by simply beating semi-dry moss surfaces and cushions on standing and fallen trees. Moss and leaf litter sifting technique was used to concentrate the samples and thus speed up the extraction process. However, Berlese funnels usually require electricity and a suitable room to accommodate them. In this study, we test the ethanol trap for collecting moss and leaf litter inhabiting flea beetles. This method may enhance our abilities to collect these groups of flea beetles and contribute to revealing their diversity and biology.

## Materials and methods

### Morphological and taxonomic methods

Observations of the habitus and diagnostic characters of flea beetles were made using the Nikon SMZ645 stereomicroscope and Nikon OPTIPHOT microscope. Genitalia with the last few abdominal tergites were separated using sharp insect pins attached to plastic sticks. The tissues surrounding the aedeagus were cleared. Female genitalia and accompanying structures (the last tergites) were immersed in a hot 10% NaOH solution for 30 s (or the appropriate time required to soften irrelevant tissue). The extra tissues surrounding the genitalia were carefully removed using insect pins. For photography, the female genitalia were mounted on slides with glycerine; male genitalia were glued to paper card points. Digital images were taken with a Canon D800 camera attached to Canon MP-E 65-mm lens or microscope lens.

Morphological terminology follows Ruan et al. (2019). Specimen labels are cited verbatim. Ninety-two specimens were assembled for this study based on museum collections and our fieldwork. Abbreviations for insect collections. **IZCAS**: Institute of Zoology, Chinese Academy of Sciences, Beijing, China. **SZPT**: Plant Protection Research Center, Shenzhen Polytechnic, Shenzhen, Guangdong, China. Field-collected and lab-reared specimens are deposited in SZPT and IZCAS.

### Rearing methods

*Benedictusfuanensis* sp. nov. were reared and observed in the laboratory environment. Rearing methods mainly follow those used for *Cangshanalticafuanensis* Ruan, Konstantinov & Damaška, 2022 (see Ruan et al. 2020). Transparent plastic rearing containers (15 cm × 7 cm × 5 cm) were selected and placed in a north-facing room to avoid direct sunlight. Two small openings were carved and sealed with non-woven fabrics, allowing for air to circulate and preventing other organisms from coming into the container. A thick layer of moist paper towel was placed at the bottom of the container to maintain proper humidity and avoid larvae from drowning in water drops; a thin layer of soil was placed above the paper towel to provide nutrition for the host plant; fresh host plant moss was collected and placed loosely above the soil layer. Distilled water was sprayed on the moss once a day to maintain humidity using a small spraying device.

### Ethanol traps

Two types of ethanol pan traps (as ethanol traps hereinafter) were used: a regular one to collect dead specimens (Fig. 8A–C) and a modified one for collecting live specimens (Fig. 8D, E). The modified trap consists of the following components: 1) a plastic container such as a plate or a bowl; 2) ethanol dipped sponge (or paper towel) placed on the bottom of the container; 3) the upper opening of the bowl is sealed by plastic film leaving a narrow opening in the middle for beetles to crawl in.

The plastic film forms a slope with a central opening at the bottom. Usually, the flea beetles would either stay close to the ethanol-dipped sponge or be trapped at the higher part of the plastic film.

Ethanol traps were usually placed close to concentrations of moss, leaf litter, or liverworts. Sometimes moss or leaf litter on the ground was slightly excavated to accommodate the ethanol traps.

Test 1. In this test, 35 modified ethanol traps (Fig. 8D, E) were placed in three moist and moss-abundant sites in a village near Fuan City, Fujian Province. Each site is approximately 100 m^2^. The experiment lasted 26 days (including five rainy days) in January and February 2021. The ethanol traps were refreshed, and the specimens were collected each day. The number of flea beetles collected was counted each day.

Test 2. In this test, 37 traditional ethanol traps (Fig. 8A–C) were used. They were placed in three sites in the Che-ba-ling nature reserve, Guangdong Province, in June 2021. The ethanol traps were refreshed, and the specimens were collected each day. The experiment lasted for three days (including one rainy day).

## Results

### Taxonomy

#### Genus Benedictus

Taxon classificationAnimaliaColeopteraChrysomelidae

Scherer, 1969

8F5B3B5C-8838-5B2F-82E5-0D61A29E1EF9

Benedictus Scherer, 1969: 99. Type species: Benedictuselisabethae Scherer, 1969, by original designation.
Himalalta Medvedev, 1990: 42. Type species: Himalaltabrevicornis Medvedev, 1990 (= Benedictusleoi Scherer, 1989). Synonymised by Sprecher-Uebersax et al. 2009: 476.

##### Distribution.

China (Fujian, Guangdong, Hongkong, Sichuan, Yunnan, Tibet), India, Nepal, Thailand, Philippines, Papua New Guinea.

### Key to Chinese *Benedictus* species

**Table:** 

1	Elytral punctures shallow and tiny, arranged in barely perceptible striae	***B.sichuanensis* Sprecher-Uebersax et al., 2009**
–	Elytral punctures deep and large, arranged in well-developed striae	**2**
2	Transverse antebasal groove of pronotum poorly defined, shallow, barely visible, and without large punctures	**3**
–	Transverse antebasal groove of pronotum well defined and deep; if shallow, then marked by a row of much deeper and larger punctures	**5**
3	Pronotum and elytra dark chestnut-brown, apex of aedeagus broadly rounded	***B.kurbatovi* Sprecher-Uebersax et al., 2009**
–	Pronotum pale brown or yellowish; elytra usually as pale as pronotum, but sometimes slightly darker; apex of aedeagus narrow, not broadly rounded	**4**
4	Ventral surface of aedeagus with relatively sharp ridge stretching from basal opening to apical 2/3	***B.belousovi* Sprecher-Uebersax et al., 2009**
–	Ventral surface of aedeagus without ridge stretching from basal opening to apical 2/3	***B.cangshanicus* Sprecher-Uebersax et al., 2009**
5	Transverse antebasal groove of pronotum shallow, marked by a row of much deeper and larger punctures	***B.wangi* sp. nov.**
–	Transverse antebasal groove of pronotum deep, well defined	**6**
6	Body bicoloured, pronotum yellowish to pale brown, head and elytra dark brown	***B.kabaki* Sprecher-Uebersax et al., 2009**
–	Body unicolorous	**7**
7	In ventral or dorsal view, apex of aedeagus wide and emarginate at middle; four dark maculations present on the abdominal tergites (Figs 4G, 5A, B), which are visible through elytra when the beetle is alive	***B.quadrimaculatus* sp. nov.**
–	In ventral or dorsal view, apex of aedeagus not wide or emarginate at middle; abdominal tergites without dark maculations	**8**
8	Apex of aedeagus sagittalis	***B.sagittalis* Damaška & Aston, 2019**
–	Apex of aedeagus not sagittalis	**9**
9	In ventral view, sides of aedeagus parallel from base to apical fourth; in lateral view, aedeagus straight at middle part, curved ventrad at basal and apical fourth, apex very slightly bent dorsad	***B.tibetanus* Sprecher-Uebersax et al., 2009**
–	In ventral view, sides of aedeagus slightly convex, widest at middle; in lateral view, aedeagus not straight at middle part	**10**
10	Head without longitudinal impression above supracallinal sulci. In ventral view, sides of aedeagus slightly and evenly convex from base to near apex, middle part not prominently wider than base; in lateral view, aedeagus evenly curved ventrad, apex not bending ventrad	***B.nigrinus* Sprecher-Uebersax et al., 2009**
–	Head with two short longitudinal impressions above supracallinal sulci. In ventral view, sides of aedeagus not evenly convex, with middle part prominently wider than base; in lateral view; aedeagus strongly curved ventrad at basal half, nearly straight at apical half, apex very slightly bent ventrad	***B.fuanensis* sp. nov.**

#### Benedictus fuanensis

Taxon classificationAnimaliaColeopteraChrysomelidae

Ruan & Konstantinov sp. nov.

2026DC66-4CFE-55AE-BFB9-6087EB458498

https://zoobank.org/6F2A1FBE-6CCC-42C0-B2A8-37196C16A07D

Figs 1 , 2 , 3

##### Type material.

***Holotype***: ♂ (SZPT), labels: 1) China, Fujian Prov., Fuan (福安), Shuyang (枢洋), 290 m, 27.1578°N, 119.6809°E, site1, 25.I–21.II.2021, Leg. Ruan, Ethanol-traps nr. moss; 2) HOLOTYPE *Benedictusfuanensis* sp. nov. Des. Ruan et al. 2022.

***Paratypes*** (72 specimens): 21♂16♀ (SZPT; some would be transferred to IZCAS), labels: 1) China, Fujian Prov., Fuan (福安), Shuyang (枢洋), 290 m, 27.1578°N, 119.6809°E, site1, 25.I–21.II.2021, Leg. Ruan, Ethanol-traps nr. moss; 2) PARATYPE *Benedictusfuanensis* sp. nov. Des. Ruan et al. 2022. • 10♂7♀ (SZPT), labels: 1) China, Fujian Prov., Fuan (福安), Shuyang (枢洋), 300 m, 27.1573°N, 119.6812°E, site2, 25.I–21.II.2021, Leg. Ruan, Ethanol-traps nr. moss; 2) PARATYPE *Benedictusfuanensis* sp. nov. Des. Ruan et al. 2022. • 3♂2♀ (SZPT), labels: 1) China, Fujian Prov., Fuan (福安), Shuyang (枢洋), 320 m, 27.1599°N, 119.6774°E site3, 25.I–21.II.2021, Leg. Ruan, Ethanol-traps nr. moss; 2) PARATYPE *Benedictusfuanensis* sp. nov. Des. Ruan et al. 2022. • 2♂5♀ (SZPT), labels: China, Fujian Prov., Fuan (福安), Shuyang (枢洋), 290 m, 27.1611°N, 119.6763°E, 13-II-2020, Extracted from moss, Leg. Y. Ruan; 2) PARATYPE *Benedictusfuanensis* sp. nov. Des. Ruan et al. 2022. • 1♂ (SZPT), labels: China, Fujian Prov., Fuan, Shuyang, 16-VIII-2019, unknown moss, Leg. Y. Ruan; 2) PARATYPE *Benedictusfuanensis* sp. nov. Des. Ruan et al. 2022. • 4♂1♀ (SZPT), labels: Guangdong, Shaoguan, Chebaling nature reserve, Luzidong, V.30-VI.4.2021, 24.6979°N, 114.1758°E, 600 m, Leg. Yongying Ruan; 2) PARATYPE *Benedictusfuanensis* sp. nov. Des. Ruan et al. 2022.

##### Diagnosis.

This new species may be distinguished from other known species of *Benedictus* by the following combination of characters: pronotum strongly convex; aedeagus widest at middle in ventral view; two longitudinal impressions present above supracallinal sulci; the facial part of the head strongly elongated; tormae of labrum (Fig. 2C) extremely long, ~ 3.5× as long as visible part of labrum.

**Figure 1.**
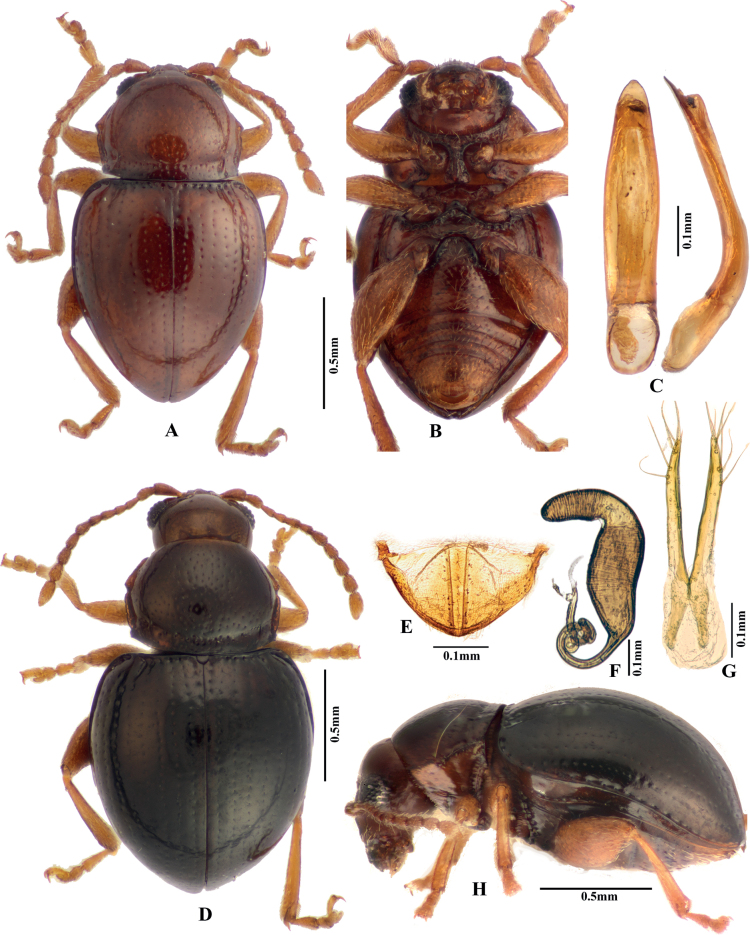
Adult morphology of *Benedictusfuanensis* sp. nov. **A** holotype, male, dorsal view **B** holotype, male, ventral view **C** median lobe of aedeagus (holotype), ventral and lateral views **D** female (paratype), dorsal view **E** last visible abdominal tergite of female **F** spermatheca **G** vaginal palpi **H** female (paratype), lateral view.

**Figure 2.**
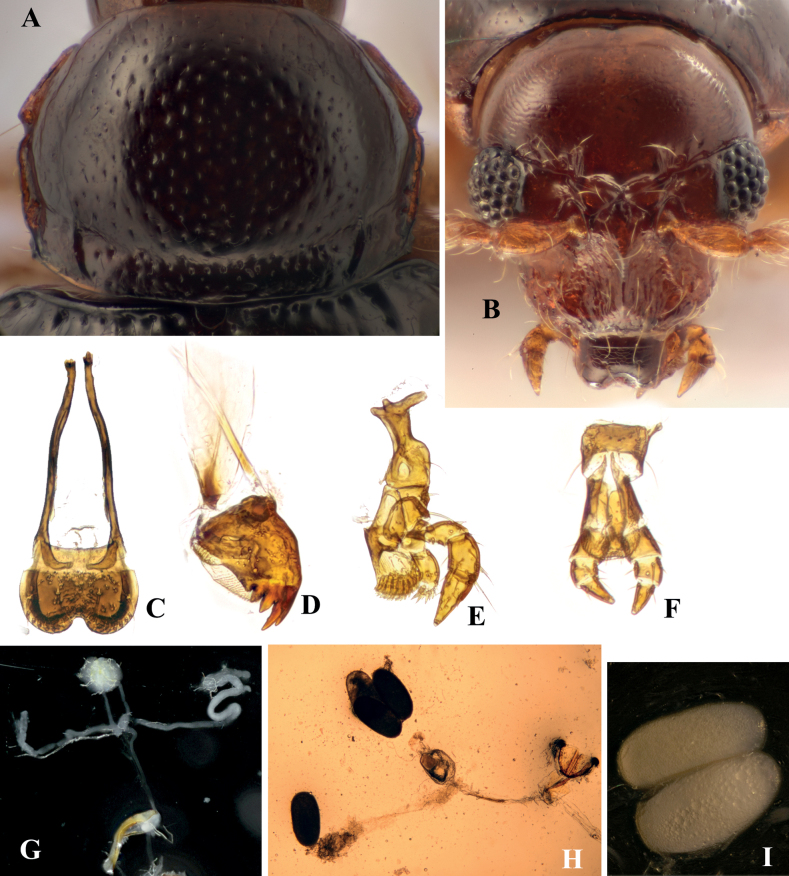
Adult morphology of *Benedictusfuanensis* sp. nov. **A** pronotum, female **B** head, female **C** labrum, showing the extremely long tormae **D** mandible **E** maxilla **F** labium **G** male reproductive system **H** female reproductive system, four eggs are visible **I** immature eggs in the ovary.

##### Description.

Male body length 1.30–1.60 mm, width 0.80–0.90 mm; female body length 1.40–1.70 mm, width 0.90–1.00 mm (measured for all type specimens). Ratio of body length to body width: 1.55–1.78 (measured in one male and one female). Dorsum yellow-brown to chestnut-brown. Venter slightly paler than dorsum. Antennae and legs uniformly pale yellow-brown to yellow-brown. Legs and antennae covered with yellow setae.

***Head*.** Head hypognathous. Vertex smooth, with very shallow reticulation; a few punctures bearing setae present above supraorbital sulci on each side; two short longitudinal impressions present at mesal side of punctures above supracallinal sulci. Antennal calli well delimited, triangular, with flattened surface. Supracallinal and supraorbital sulci deep, forming oblique straight line. Supra-antennal sulcus poorly developed. Facial part of head strongly elongated. Frontal ridge widest between antennal sockets, much narrowed and ridged towards clypeus; each side of frontal ridge concave and looks coarse being covered with minute longitudinal ridges. Fronto-genal ridge present. Labrum with two pairs of setae, deeply emarginate on anterior margin. Mandibles symmetrical, palmate; each mandible with five sharp teeth, mesal side with a membranous lobe bearing dense microtrichia. Tormae of labrum extremely long, ~ 3.5× as long as visible part of labrum. Proportions of antennomere lengths: 100: 56: 45: 33: 47: 42: 54: 53: 56: 58: 87 (measured in one individual).

***Thorax*.** Pronotum strongly convex, ratio of pronotum width (measured at posterior edge) to length: 1.37–1.42 (measured in two males and two females). Pronotum widest at posterior part of anterolateral callosity. Anterolateral callosity well-developed, elongate, and straight, with an anterolateral setiferous pore situated at posterior end. Procoxal cavities open posteriorly. Base of pronotum with deep and transverse antebasal groove, delimited by well-developed longitudinal grooves on each side.

Elytra strongly convex, humeral calli absent. Elytra with punctures arranged in regular lines. Hind wings absent.

***Legs*.** First male protarsomere larger than that of female. Length of metatibia to first metatarsomere in male: 100: 31.

***Male genitalia*.** Median lobe of aedeagus in ventral view: widest at middle, ventral surface smooth, sides narrowing from middle to apex; apex narrowly rounded, without denticle. Median lobe of aedeagus in lateral view: widest at base, strongly curved ventrad at basal half, apical half nearly straight, with apex very slightly bent ventrad.

***Female genitalia*.** Spermathecal pump cylindrical, very slightly curved, apex broad and rounded; without clear border with receptacle; more or less perpendicular to receptacle. Receptacle of spermatheca cylindrical, gradually narrowed towards spermathecal duct, with sides slightly curved near middle. Spermathecal duct has coils.

##### Variation.

In the specimens collected from Fujian province, males have a paler colour than females; males vary slightly in body size; females have more or less invariable body size. In the specimens from Guangdong province, males have a deeper colour than females.

##### Etymology.

This species is named after the type locality, Fuan city; the name also indicates that the species is sympatric with *Cangshanalticafuanensis*Ruan et al. (2022). The specific epithet is a noun in apposition.

##### Type locality.

Shuyang, Fuan, Fujian Prov., China.

##### Distribution.

China (Fujian, Guangdong).

##### Host plant.

*Benedictusfuanensis* sp. nov. primarily fed on *Hypnumplumaeforme* Wilson (Hypnaceae) in the laboratory environment. They were spotted on *H.plumaeforme* at night in the type locality. We found they also feed on Racopilumcf.aristatum when there is no *H.plumaeforme* present in the rearing container.

##### Biology.

Forty live individuals were collected by modified ethanol traps (Fig. 8D, E) and reared in a plastic container in the laboratory. Rearing methods are the same as those used for *Cangshanalticafuanensis*, follows Ruan et al. (2020). Copulation was observed frequently in the lab-reared individuals of *B.fuanensis*; in some cases, a single copulation could last for more than 24 hours, with the male constantly staying on the back of the female. The rearing lasted for 46 days; however, no eggs or larvae were found. This means the biological habits of *B.fuanensis* may be slightly different from those of *Cangshanalticafuanensis*.

**Figure 3.**
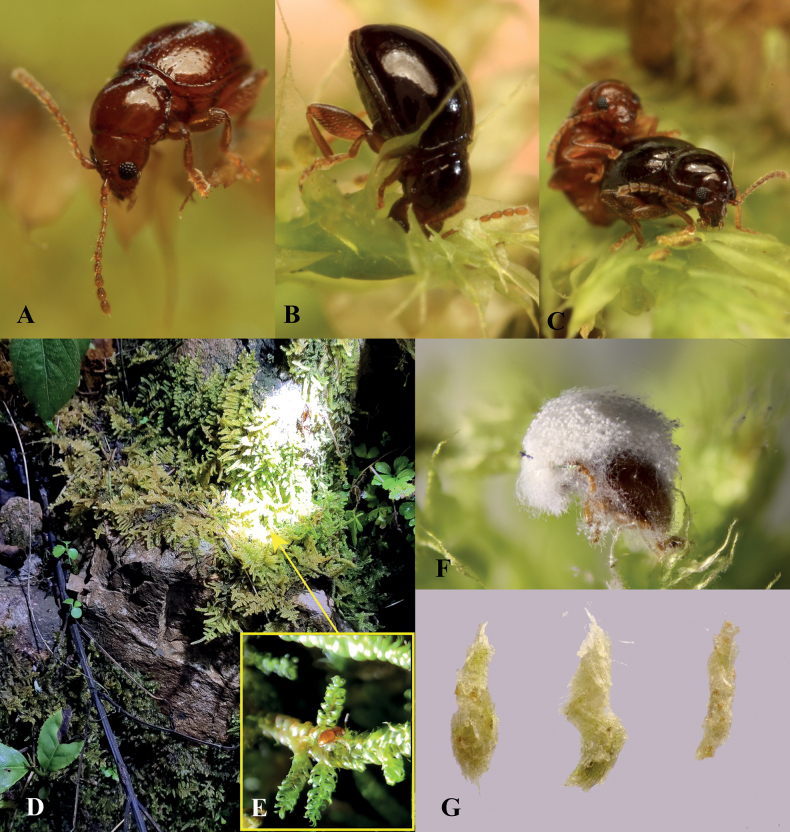
Biology of *Benedictusfuanensis* sp. nov. **A** male **B** female adult feeding on the top of a young shoot of the *Hypnumplumaeforme* Wilson **C** male and female *in copula***D** habitat at the type locality, photographed at night **E** an individual discovered on the host plant at night at the type locality **F** an individual reared in the lab infected by fungi **G** faeces of individuals reared in the lab.

*Benedictusfuanensis* sp. nov. and *Cangshanalticafuanensis* were found on the same host plant in the same moss cushion in Fuan, Fujian Province. They are also quite similar in some biological characteristics. For instance, adults of both species were usually discovered on the surface of *Hypnumplumaeforme* Wilson at night with high humidity; they both like to feed on the top of the young shoots of the host plant, so that the ends of young shoots are usually chopped off by beetle feeding, which is destructive to the host plant; the faeces of their larvae and adults mainly consist of undigested fragments of host plant leaf (see Fig. 3G). The interaction of the two species in nature is still unknown.

*Benedictusfuanensis* sp. nov. also has large eggs, small egg numbers, and fewer ovarioles. These features are similar to those of *Cangshanalticafuanensis*. Based on the dissection of three female specimens, four to six eggs could be found inside a female abdomen. Egg length 0.60–0.62 mm; width 0.25–0.31 mm (measured on two eggs); egg length equals ~ 40% of female body length.

The jumping ability of two individuals was tested. The horizontal jumping distance ranged from 3.5 cm to 11.7 cm. *Benedictusfuanensis* sp. nov. has far less explosive jumps compared to *Cangshanalticafuanensis*.

#### Benedictus quadrimaculatus

Taxon classificationAnimaliaColeopteraChrysomelidae

Ruan & Konstantinov sp. nov.

37F4BFA3-774E-5722-A1DB-AC249040C466

https://zoobank.org/86DA5B90-EA05-4008-B22F-6DC74CF9C8CF

Figs 4 , 5

##### Type material.

***Holotype***: ♂ (SZPT), labels: 1) China, Yunnan, Yuanyang County, Xinjie, 23.1163°N, 102.7690°E, 1900 m. Leg. Y. Ruan & M. Zhang 2019.VII.28, Extracted from moss; 2) HOLOTYPE *Benedictusquadrimaculatus* sp. nov. Des. Ruan et al. 2022.

***Paratypes***: 6♂6♀ (SZPT; some would be transferred to IZCAS), labels: 1) China, Yunnan, Yuanyang County, Xinjie, 23.1163°N, 102.7690°E, 1900 m. Leg. Y. Ruan & M. Zhang 2019.VII.28, Extracted from moss; 2) PARATYPE *Benedictusquadrimaculatus* sp. nov. Des. Ruan et al. 2022.

##### Diagnosis.

**Figure 4.**
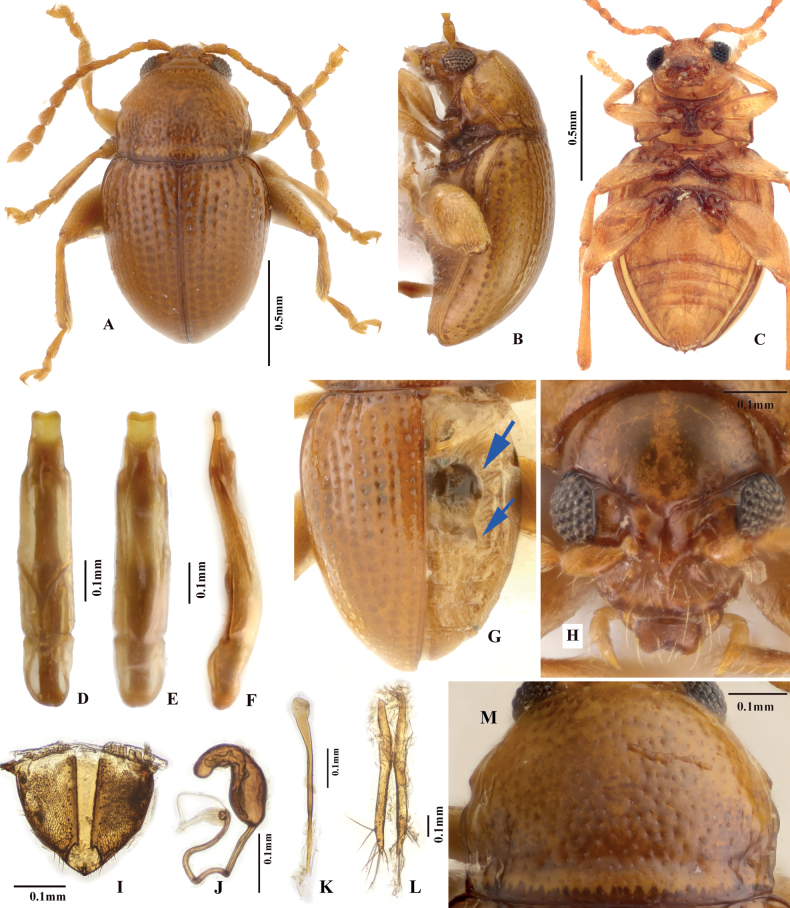
Adult morphology of *Benedictusquadrimaculatus* sp. nov. **A–C** holotype, dorsal, lateral, and ventral views **D–F** median lobe of aedeagus (holotype), ventral, dorsal, and lateral views **G** sclerotised and darkened area on the abdominal tergites (arrowed), which are visible through elytra as black spots when the beetle is alive **H** head **I** last visible abdominal tergite of female **J** spermatheca **K** tignum **L** vaginal palpi **M** pronotum.

**Figure 5.**
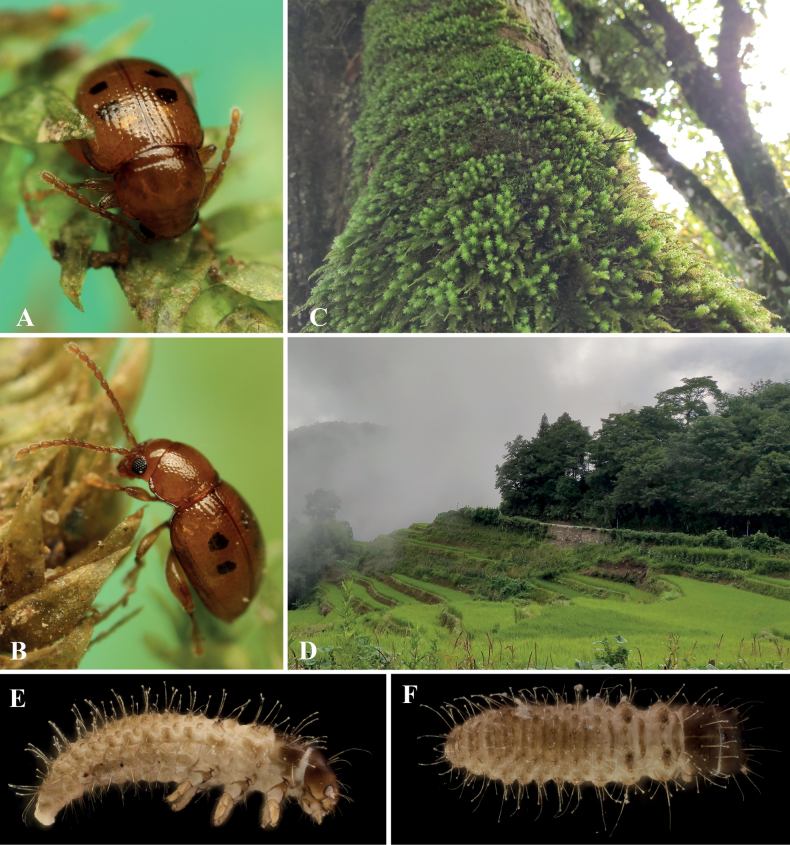
Biology of *Benedictusquadrimaculatus* sp. nov. **A, B** photography of living individuals in lab environment **C** photography of the moss cushion at the type locality **D** habitat environment near the type locality **E, F** habitus of two unknown larvae extracted along with the adults of *B.quadrimaculatus* from moss.

This new species may be distinguished from other known species of *Benedictus* by the following combination of characters: in ventral or dorsal view, apex of median lobe of aedeagus wide and emarginate at middle; four dark maculations present on the abdominal tergites (Figs 4G, 5A, B), which are more prominent when the beetle is alive; antennal calli subquadrate with a fovea present between them. Black spots on the abdominal tergites that are visible through elytra is a highly unusual feature that we have not observed in flea beetles before.

##### Description.

Male body length 1.35–1.45 mm, width 0.80–0.85 mm; female body length 1.45–1.50 mm, width 0.80–0.85 mm (based on all type specimens). Ratio of body length to body width: 1.70–1.77 (one male and one female measured). Entire body evenly yellow-brown to chestnut-brown, including antennae and legs.

***Head*.** Head hypognathous. Vertex smooth, without reticulation; a few punctures bearing setae situated above supraorbital sulci on each side. Antennal calli well delimited, subquadrate, and slightly convex; fovea present between antennal calli. Supracallinal and supraorbital sulci deep, forming oblique straight line. Supra-antennal sulcus poorly developed. Facial part of head relatively short. Frontal ridge widest between antennal sockets, strongly narrowed and ridged towards clypeus; frons concave and smooth on each side of frontal ridge, surface without minute longitudinal ridges. Proportions of antennomere lengths: 100: 64: 45: 45: 66: 53: 72: 78: 73: 78: 110 (measured in one individual).

***Thorax*.** Pronotum moderately convex, ratio of pronotum width (measured at middle) to length: 1.30–1.42 (measured in one male and one female). Pronotum widest at middle part. Anterolateral callosity strongly developed, elongate, and somewhat straight, with anterolateral setiferous pore situated at posterior end. Procoxal cavities open posteriorly. Base of pronotum with deep and transverse antebasal groove bearing coarse and large punctures; transverse antebasal groove delimited by a well-developed longitudinal groove on each side.

Elytra convex, humeral calli absent. Elytra with punctures arranged in regular lines. Hind wings absent.

***Legs*.** First male protarsomere larger than that of female. Length of metatibia to first metatarsomere in male: 100: 30.

***Male genitalia*.** Median lobe of aedeagus in ventral view: widest at middle; ventral surface smooth; sides parallel from base to apical fourth, abruptly narrowed with a step at apical fourth; apex wide, emarginated in middle, without denticle. Median lobe of aedeagus in lateral view: slightly sinuate, curved ventrad at basal 3/4, bent dorsad at apical 1/4, apex straight.

***Female genitalia*.** Spermathecal pump cylindrical, very slightly curved, apex broad and rounded; without clear border with receptacle; more or less perpendicular to receptacle. Receptacle of spermatheca pear-shaped, with sides convex. Spermathecal duct without coils.

##### Variation.

The shape of the pronotum varied slightly by having slightly lesser widths and straighter lateral sides in some individuals.

##### Etymology.

This species is named after the four dark maculations on its abdominal tergites (Fig. 4G), which are prominent when the beetle is alive (Fig. 5A, B).

##### Type locality.

Yuanyang County, Yunnan Prov., China.

##### Distribution.

China (Yunnan).

##### Host plant.

Unknown.

##### Biology.

This species is extracted from moss cushions containing multiple moss species using a modified fan-driven Berlese funnel (see Ruan et al. 2020). Live individuals were reared in the laboratory environment; however, no feeding behaviour was observed.

Although two larvae (Fig. 5E, F) were extracted along with the adults from moss, it is unknown if they are conspecific.

#### Benedictus wangi

Taxon classificationAnimaliaColeopteraChrysomelidae

Ruan & Konstantinov sp. nov.

E66764D3-8259-50E6-9C39-0DE73A24A6B2

https://zoobank.org/0019D11C-D497-4805-A567-4382902120DA

Fig. 6

##### Type material.

***Holotype***: ♂ (IZCAS), labels: 1) Tibet, Linzhi, Milin county, Sejilashan, 318 km, 4174 km, 29°38'15.37"N, 94°42'52.89"E, 4106 m, from the soil under rhododendron, 2016-VI-13, Leg. Yi Wei; 2) HOLOTYPE *Benedictuswangi* sp. nov. Des. Ruan et al. 2022.

***Paratypes***: 2♂3♀ (SZPT), labels: 1) Tibet, Linzhi, Milin county, Sejilashan, 318 km, 4174 km, 29°38'15.37"N, 94°42'52.89"E, 4106 m, from the soil under rhododendron, 2016-VI-13, Leg. Yi Wei; 2) PARATYPE *Benedictuswangi* sp. nov. Des. Ruan et al. 2022. [Part of the paratype materials will be transferred to IZCAS]

**Figure 6.**
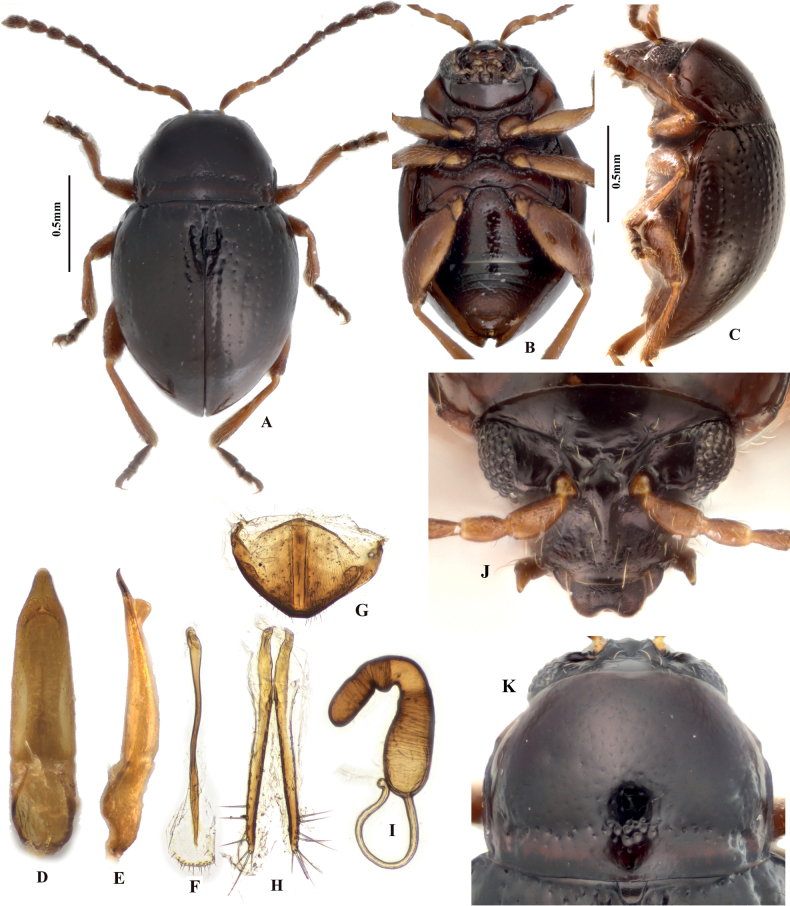
Adult morphology of *Benedictuswangi* sp. nov. **A–C** holotype; dorsal, ventral, and lateral views **D, E** median lobe of aedeagus (holotype), ventral, and lateral views **F** tignum **G** last visible abdominal tergite of female **H** vaginal palpi **I** spermatheca **J** head **K** pronotum.

##### Diagnosis.

This new species may be distinguished from other known *Benedictus* species by the following combination of characters: a line of deep and large punctures present on the antebasal groove of pronotum; spermathecal pump has a bulge at base; in lateral view, median lobe of aedeagus almost straight, only slightly curved ventrad near apex.

This new species is close to those *Benedictus* species that have a broad pronotum without a constriction near the base, such as *B.nobding* Sprecher-Uebersax, Konstantinov, Prathapan & Döberl, 2009; *B.thumsila* Sprecher-Uebersax, Konstantinov, Prathapan & Döberl, 2009; *B.yatongla* Sprecher-Uebersax, Konstantinov, Prathapan & Döberl, 2009; *B.lauribina* Sprecher-Uebersax, Konstantinov, Prathapan & Döberl, 2009, and *B.kurbatovi* Sprecher-Uebersax, Konstantinov, Prathapan & Döberl, 2009. This species could be distinguished from all these by having a line of deep and large punctures on the transverse antebasal groove of the pronotum.

This new species is especially close to *Benedictuslauribina* in the general shape of the body and spermatheca. However, it can be differentiated from *B.lauribina* by the following characters: the body colour is chestnut-brown to dark brown, the apex of aedeagus is broadly rounded, and a line of deep and large punctures present on the transverse antebasal groove of the pronotum. While in *B.lauribina*, the body colour is yellow-brown, the apex of aedeagus is acute, and there are no deep and large punctures on the transverse antebasal groove of the pronotum.

##### Description.

Male body length 1.55–1.65 mm, width 0.95–1.05 mm; female body length 1.75–1.80 mm, width 1.09–1.11 mm (based on all type specimens). Ratio of body length to width: 1.59–1.63 (measured in one male and one female). Entire body evenly chestnut-brown to deep brown; antennae and legs yellow-brown to chestnut-brown.

***Head*.** Head hypognathous. Vertex smooth, without reticulation, a few punctures bearing setae present above supraorbital sulci on each side. Antennal calli well delimited, triangular, slightly convex; fovea present between antennal calli. Supracallinal and supraorbital sulci deep, forming nearly straight line. Supra-antennal sulcus poorly developed. Facial part of head slightly elongated. Frontal ridge widest between antennal socket, strongly narrowed and ridged towards clypeus; frons concave on each side of frontal ridge, surface without minute longitudinal ridges. Proportions of antennomere lengths: 100: 64: 58: 58: 66: 62: 75: 64: 65: 70: 107 (measured in one individual).

***Thorax*.** Pronotum moderately convex, ratio of pronotum width (measured at middle) to length: 1.36–1.39 (measured in one male and one female). Pronotum widest at middle. Anterolateral callosity poorly developed. Procoxal cavities open posteriorly. Base of pronotum with deep and transverse antebasal groove bearing coarse and large punctures, delimited by well-developed longitudinal grooves on each side.

Elytra convex, humeral calli absent. Elytra with punctures arranged in regular lines. Hind wings absent.

***Legs*.** First male protarsomere only slightly larger than that of female. Length of metatibia to first metatarsomere in male: 100: 28.

***Male genitalia*.** Median lobe of aedeagus in ventral view: widest at basal third; ventral surface smooth; sides parallel from basal half, gradually narrowed apically; apex widely rounded, without denticle. Median lobe of aedeagus only slightly curved in lateral view: straight at basal 2/3, slightly curved ventrad at apical 2/3, apical end bent dorsad.

***Female genitalia*.** Spermathecal pump cylindrical, apex broad and rounded; without clear border with receptacle; make acute angle with receptacle. Receptacle of spermatheca more or less cylindrical, with sides slightly convex.

##### Variation.

No prominent variation was observed.

##### Etymology.

The specific name is after the Chinese entomologist and flea beetle specialist Mr. Shuyong Wang.

##### Type locality.

Linzhi, Tibet, China.

##### Distribution.

China (Tibet).

##### Host plant.

Unknown.

### Testing of the ethanol traps for collecting leaf litter and moss-inhabiting flea beetles

#### Test 1 (Figs 7, 8D, E; see Materials and methods section for more information)

The number of specimens collected presents a positive correlation with the temperature. The efficiency of the ethanol traps was highly affected by the lower air temperature and the rain in winter, which may reduce the activity of the flea beetles. In total, 122 individuals of moss or liverwort-feeding flea beetles were collected: 82 individuals of *Benedictusfuanensis* sp. nov., 19 individuals of *Cangshanalticafuanensis*, and 21 individuals of *Minota* sp. The data show that four or five individuals of moss or liverwort-feeding flea beetles were collected each day; on average, every eight ethanol traps yield one individual each day.

Except for those flea beetle species mentioned above, four individuals of *Chaetocnemaconstricta*Ruan et al. 2014, and one individual of *Longitarsus* sp. were also discovered in the traps. Many other insects were also found in the trap, and this method may also be useful to collect other moss and leaf litter inhabiting beetles.

#### Test 2 (Fig. 8A–C; see Material and methods section for more informtion)

In total, 39 individuals of moss-, liverwort-, or leaf litter-inhabiting flea beetles were collected. They belong to three flea beetle genera: *Benedictus*, *Minota* Kutschera, and *Clavicornaltica* Scherer. The data show that 13 specimens could be collected each day; on average, approximately every three ethanol traps yield one flea beetle each day.

**Figure 7.**
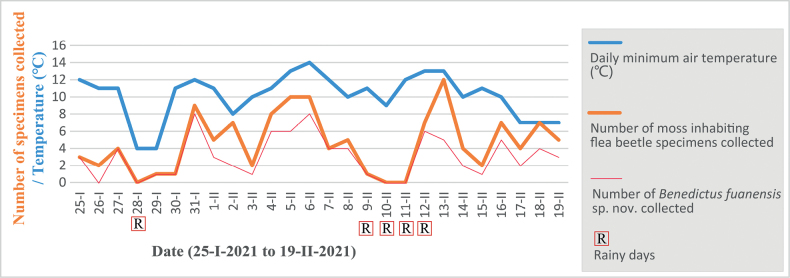
Number of moss-inhabiting flea beetles collected by ethanol traps influenced by the weather. The lowest air temperature of each day (provided by the local weather bureau) is marked in the blue line; the number of all moss-inhabiting flea beetle specimens collected each day is marked in the orange line; the number of *Benedictusfuanensis* sp. nov. is marked by the red line. The letter R in a red box indicates rainy days. The figure shows that lower temperatures and rainy weather highly reduce the efficiency of the ethanol traps.

**Figure 8.**
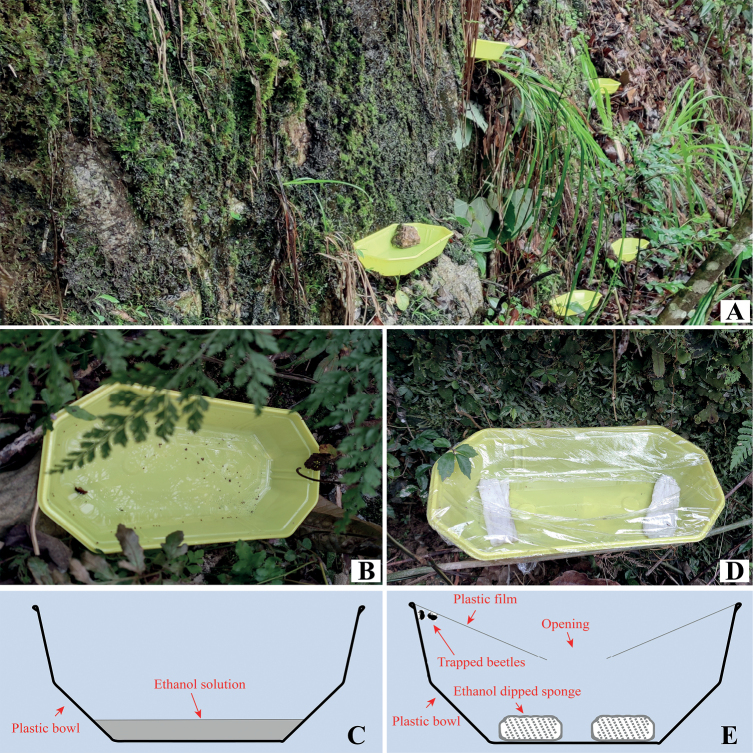
Ethanol traps used for collecting leaf litter and moss-inhabiting flea beetles **A–C** regular ethanol traps were placed close to moist moss, liverwort, or leaf litter to collect flea beetles **D, E** modified ethanol trap for collecting living individuals; diagram in inset **E** shows that ethanol-dipped sponge is used as bait, the upper opening of the bowl is sealed by plastic film leaving a narrowing opening for beetles to crawl in, the plastic film forms a slope; when the flea beetles try to escape, they usually crawl upwards and could be trapped by the slope.

## Discussion

Counting three new species described in this work, there are now 11 *Benedictus* species known from China and 29 species from the world. The previously reported *Benedictus* species all inhabit middle to high altitudes (based on published works). However, the discovery of *Benedictusfuanensis* sp. nov. shows that they also adapt to low-elevation (290–320 m) environments.

It is rather intriguing that *Benedictusfuanensis* sp. nov. and *Cangshanalticafuanensis* are not only sympatric but also share the same host plant. They are also similar in feeding on the top of the young shoots of the host plant and having small number of large eggs and fewer ovarioles. These may be related to the miniaturisation of their body size. The rearing environment of *Benedictusfuanensis* sp. nov. was maintained similarly to that in the rearing of *C.fuanensis* (Ruan et al. 2020). The failure to produce the second generation implies that the microenvironment they adapt to may differ slightly from that of *C.fuanensis*. The high humidity maintained in the rearing process of *C.fuanensis* may not be optimal for *Benedictusfuanensis* sp. nov.

The ethanol traps were tested and proven quite efficient in collecting moss- and leaf litter-inhabiting flea beetles. However, it is uncertain if ethanol works as bait for the beetles, which needs to be tested in future field works.

## Supplementary Material

XML Treatment for Benedictus

XML Treatment for Benedictus fuanensis

XML Treatment for Benedictus quadrimaculatus

XML Treatment for Benedictus wangi

## Additional information

### Author ORCIDs

Yongying Ruan https://orcid.org/0000-0002-5025-5592

Alexander S. Konstantinov https://orcid.org/0000-0001-6578-6735

Albert F. Damaška https://orcid.org/0000-0002-3640-626X

Jun Chen https://orcid.org/0000-0002-0325-2532

### Data availability

All of the data that support the findings of this study are available in the main text or Supplementary Information.
